# Usefulness of intraoperative optical coherence tomography to minimize the intraocular lens tilt during the intrascleral fixation: a clinical and experimental evaluation

**DOI:** 10.1038/s41598-023-39294-0

**Published:** 2023-07-26

**Authors:** Yasuyuki Sotani, Hisanori Imai, Yukako Iwane, Hiroko Yamada, Wataru Matsumiya, Akiko Miki, Sentaro Kusuhara, Makoto Nakamura

**Affiliations:** Department of Surgery, Division of Ophthalmology, Kobe University Graduate School of Medicine, 7-5-2 Kusunoki-Cho, Chuo-Ku, Kobe, 650-0017 Japan

**Keywords:** Lens diseases, Vision disorders

## Abstract

To report the usefulness of intraoperative real-time adjustment of intraocular lens (IOL) tilt during the intrascleral fixation with intraoperative optical coherence tomography (iOCT) as a clinical evaluation and investigate the factors contributing to IOL tilt using iOCT as an experimental evaluation. Retrospective cohort study and experimental research. As a clinical evaluation, the medical records of 43 eyes of 41 patients who underwent intrascleral IOL fixation combined with real-time iOCT observation were retrospectively reviewed. As an experimental evaluation, in order to investigate the factors contributing to IOL tilt, the four experiments were performed using iOCT. The mean IOL tilt angle (°) at the end of surgery and 3 months after surgery were 1.81 ± 1.15 and 2.10 ± 1.66, respectively (*p *= 0.46). No apparent intra- or postoperative complications occurred during the follow-up period. The experimental evaluation indicated that the IOL tilt was influenced by the insertion angle of the haptic in the vertical direction. The mean IOL tilt angle (°) was 1.94 ± 0.09, 4.67 ± 0.11, 8.90 ± 0.11, and 15.78 ± 0.85 when the insertion angle of the haptic was 0°, 10°, 27.5°, and 45° in the vertical direction, respectively (*p *< 0.01). Clinical and experimental IOL tilt assessment using iOCT is interactively useful for better quality surgery and better postoperative outcome.

## Introduction

Recently, there have been increases in the frequencies of crystalline and intraocular lens (IOL) dislocation^1^. Consequently, the number of patients requiring IOL scleral fixation has also increased. Representative types of IOL scleral fixation include IOL suturing and intrascleral IOL fixation, but postoperative problems can occur with suturing, including displacement of the IOL due to looseness or breakage of the sutures^2–4^. Therefore, in recent years, intrascleral fixation is preferred over suturing^5^.

There are various methods of intrascleral IOL fixation^6–8^, but the frequency of performance of the Yamane intrascleral fixation technique has increased because of the ease of use^9^. Follow-up studies and arrangement methods relating to the Yamane intrascleral fixation technique have all reported favorable results^10^. However, with the Yamane intrascleral fixation technique, accurate setting of the needle insertion angle is difficult, and considerable practice is needed to achieve competence. Moreover, the large IOL tilt can develop unexpectedly^11^, resulting in postoperative refractive error^11^ and postoperative ocular coma-like aberrations^12^.

Various methods can be applied for overcoming the IOL tilt with the Yamane intrascleral fixation technique, including verification of the IOL tilt by checking the Purkinje-Sanson mirror image, surgical techniques using stabilizers^13^, and adjustment based on the amount of haptic pulled-out^11^. However, irrespective of the method used, intraoperative, real-time accurate verification of the angle of IOL tilt is difficult. Moreover, the cause of IOL tilt has not been investigated in detail to date.

Intraoperative optical coherence tomography (iOCT) has recently been found useful for safe and accurate surgery and providing information that is not visible under microscopy; moreover, the application of iOCT has increasingly gained popularity^14^. Although the most effective procedure for manipulating iOCT during intrascleral IOL fixation is still under debate, one useful technique for real-time adjustment of IOL tilt using iOCT was recently reported^15^.

In this study, we followed-up the method of intraoperative real-time adjustment of the IOL tilt with iOCT^15^ as a clinical evaluation and investigated the factors contributing to IOL tilt using an iOCT system (RESCAN 700; Carl Zeiss Meditec, Oberkochen, Germany) as an experimental evaluation.

## Methods

### Clinical evaluation

In total, 43 eyes of 41 patients who underwent Yamane intrascleral IOL fixation at our hospital between March 2021 and September 2022 were included in this study. The subjects were observed for 3 months after surgery. Medical records were retrospectively analyzed. The study was conducted in accordance with the Declaration of Helsinki, and approved by the Ethics Committee of Kobe University Graduate School of Medicine (No. B210074, June 25, 2021.). The need for informed consent was waived by the committee because of the retrospective, observational design of the study. Nonetheless, patients were able to withdraw consent at any time, which could be accessed on the hospital homepage as an opt-out choice. The following parameters were extracted from the medical records and used for statistical analysis: sex, age, preoperative best-corrected visual acuity (BCVA), BCVA at 1 month after surgery, BCVA at 3 months after surgery, preoperative intraocular pressure (IOP), IOP at 1 month after surgery, IOP at 3 months after surgery, preoperative corneal endothelial cell density (CECD), CECD at 1 month after surgery, CECD at 3 months after surgery, IOL tilt angle at the end of surgery and 1 month and 3 months after surgery, surgically induced astigmatism 3 months after surgery, refractive difference from the predicted value 3 months after surgery, and occurrence of intra- or postoperative complications.

### Surgical procedure

All surgeries were performed at our hospital by one experienced vitreoretinal surgeon (H.I.). Sub-Tenon anesthesia was administered using 4 ml of mixed 2% lidocaine and 0.5% levobupivacaine. The 27-gauge pals plana vitrectomy (27GPPV) with a wide-angle non-contact viewing system (Resight®; Carl Zeiss Meditec AG, Jena, Germany) was performed using the Constellation Vision System (Alcon Laboratories, Inc., Fort Worth, TX, USA). Three cannulas were created with conjunctival displacement and oblique-angled sclerotomies in the inferotemporal, superotemporal, and superonasal quadrants 3.0–4.0 mm posterior to the limbus. For eyes without the history of vitrectomy, following core vitrectomy, the vitreous gel was visualized after injecting triamcinolone acetonide (TA) (MaQaid; Wakamoto Pharmaceutical, Tokyo, Japan) during midperipheral vitrectomy, and a complete 360° vitrectomy was performed for the peripheral vitreous gel. Posterior vitreous detachment was intentionally done if needed. After vitrectomy, for eyes with IOL dislocation, a side port corneal incision was created with a 20-gauge MVR blade (MVR-Lance; Alcon Laboratories, Inc., Fort Worth, TX USA). The dispersive viscoelastic materials (VISCOAT 0.5; Alcon Japan Ltd., Tokyo, Japan) were injected to fill the anterior chamber. Then, the IOL was positioned into the anterior chamber. A 2.4-mm bent transconjunctival single-plane sclerocorneal incision or corneal incision was made with a 2.4-mm slit knife (MSR24; Mani, Inc., Tochigi, Japan) at the 11 o’clock position. The IOL was resected in half and removed from the main incision. For eyes with crystalline lens dislocation, if it was located at the bottom of the fundus, after floating it as far as the iris surface using perfluorocarbon liquid, it was removed by phacoemulsification. If the crystalline lens was fixable using capsule expander, after fixation, it was removed by phacoemulsification and aspiration.

Next, the IOL (NX-70S; Santen Pharmaceutical Co., Ltd. Osaka, Japan) was inserted into the anterior chamber. Using a Yamane double-needle stabilizer(Geuder AG, Heidelberg, Germany) , two 30-gauge needles (TSK ultra-thin wall needle; Tochigi Seiko, Tochigi, Japan) were inserted into the sclera at the 4 and 10 o’clock meridians. Using Maxgrip-type ILM forceps(Alcon Laboratories) , the haptics of the IOL were then inserted into the lumens of the two 30-gauge needles, and pulled out for externalization. The position of the IOL was then adjusted with microscopic observation, and the dispersive viscoelastic substances were removed by irrigation and aspiration (IA) as much as possible; thereafter, TA was dispersed inside the anterior chamber. The TA inside the anterior chamber was deposited on the surface of the IOL, and iOCT (RESCAN 700) therefore displayed it as a high-intensity line. While continuing iOCT observation, the IOL tilt in the vertical and horizontal directions was adjusted, and the amount of haptic pulled-out was adjusted. Finally, after trimming the unnecessary haptics, the ends of the haptics were cauterized using an ophthalmic cautery device (Accu-Temp Cautery; Beaver Visitec, Waltham, MA, USA) to make a flange. The flange was pushed back and fixed into the scleral tunnels. The final IOL tilt was assessed intraoperatively by observing the iOCT, and the absence of an obvious tilt was confirmed. TA was then removed from the anterior chamber by IA, completing the surgery. (Supplemental Digital Content 1 demonstrates the method of intraoperative real-time adjustment of the IOL tilt with iOCT.)

### Experimental evaluation

**Figure 1:**
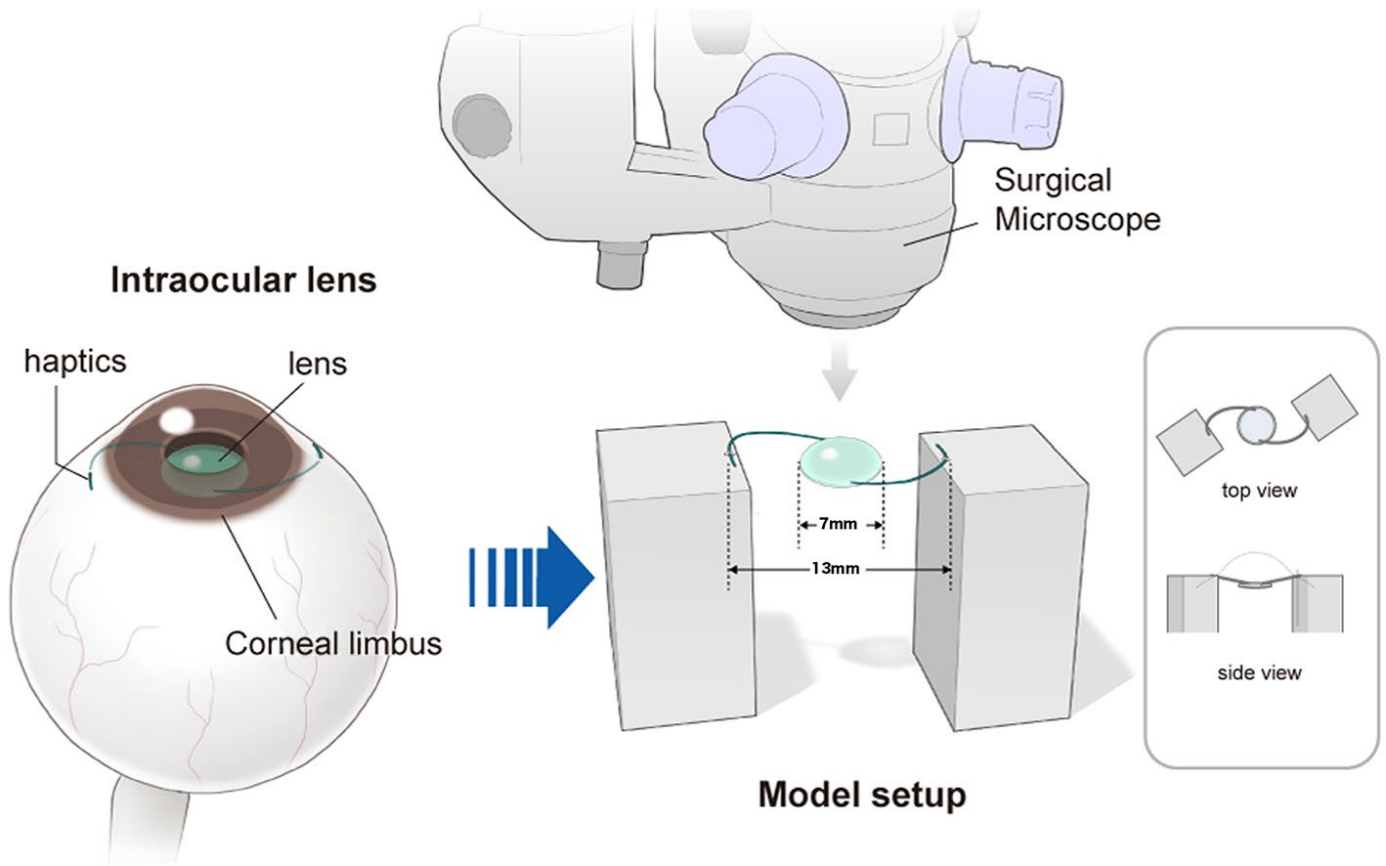
Schematic representation of the experimental evaluations. Both ends of the IOL haptic were fixed to a stand at positions 1 mm from the tip, with the optical surfaces positioned horizontally. The stand used was then positioned under the surgical microscope so that the distance between the haptic tips was 13 mm. IOL = intraocular lens.

In order to investigate the factors contributing to IOL tilt and decentration, the experiments detailed below were performed. Both ends of the IOL haptic were fixed to a stand at positions 1 mm from the tip, with the optical surfaces positioned horizontally. The stand used was then positioned under the surgical microscope (RESCAN 700) so that the distance between the haptic tips was 13 mm (Fig. 1). The inter-haptic distance was measured using the CALLISTO eye function mounted in the surgical microscope (RESCAN 700). Thereafter, the images of the IOL line were taken in vertical and horizontal directions by iOCT, because iOCT generates images in which the anterior surface line of the IOL has high intensity in the atmosphere. The IOL tilt and decentration were analyzed as follows, taking as the standard the IOL position at that time-point. (Supplemental Digital Content 2 demonstrates how the IOL is imaged and moved under iOCT during the experimental evaluation.)

- *Experiment* 1: Investigation of the effects of inconsistency in the distance from the limbus to the 30-gauge needle insertion position on tilt and decentration

**Figure 2:**
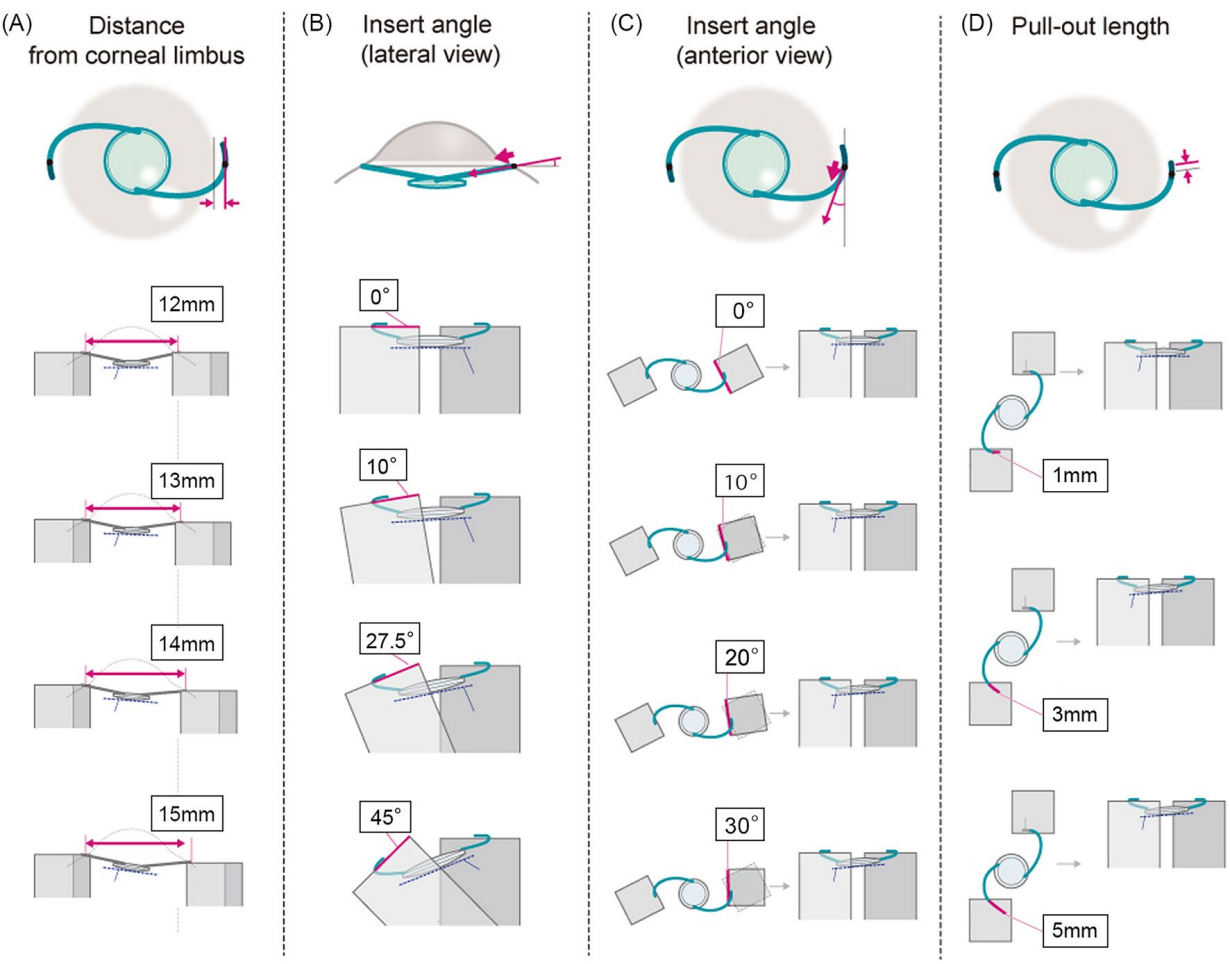
Schematic representation of experimental evaluations 1–4. (**A**) Investigation of the effects of inconsistency in the distance from the limbus to the needle insertion position on tilt and decentration. The haptic on one side was fixed, and the other haptic was moved so that the distance between the haptic tips as shown by CALLISTO photography was 12 mm, 13 mm, 14 mm, and 15 mm. (**B**) Investigation of the effects of inconsistency in the needle insertion angle in the vertical direction. With observation from the horizontal direction, the angle of the haptic on one side was set at 0°, 10°, 27.5°, and 45°, on the basis of the reference angle (0°). (**C**) Investigation of the effects of inconsistency in the needle insertion angle in the horizontal direction. With observation from above, the angle of the haptic on one side was set at 0°, 10°, 20°, and 30°, on the basis of the reference angle (0°). (**D**) Investigation of the effects of inconsistency in the amount of the IOL pulled out on tilt and decentration. The haptic on one side was fixed at 1 mm, and the haptic on the other was fixed at 1 mm, 3 mm, and 5 mm from the tip. *IOL* Intraocular lens.

The haptic on one side was fixed, and the other haptic was moved so that the distance between the haptic tips as shown by CALLISTO photography was 12, 13, 14, and 15 mm respectively. iOCT and CALLISTO photography were used for the analysis of IOL tilt and decentration at each time-point (Fig. 2A).

- *Experiment* 2: Investigation of the effects of inconsistency in the 30-gauge needle insertion angle on tilt and decentration: Investigation of inconsistency in the vertical direction.

With observation from the horizontal direction, the angle of the haptic on one side was set at 0°, 10°, 27.5°, and 45° respectively, on the basis of the reference angle (0°), and iOCT and Callisto photography were used for the analysis of IOL tilt and decentration at each time-point (Fig. 2B).

- *Experiment* 3: Investigation of the effects of inconsistency in the 30-gauge needle insertion angle on tilt and decentration: Investigation of inconsistency in the horizontal direction.

With observation from the vertical direction, the angle of the haptic on one side was set at 0°, 10°, 20°, and 30° respectively, on the basis of the reference angle (0°), and iOCT and CALLISTO photography were used for the analysis of IOL tilt and decentration at each time-point (Fig. 2C).

- *Experiment* 4: Investigation of the effects of inconsistency in the amount of IOL pulled-out on tilt and decentration.

As mentioned above, both ends of the IOL haptic were fixed to a stand 1 mm from the tip first. The stand was then placed under an operating microscope (RESCAN 700) with a distance of 13 mm between the fixed positions of the haptic. Based on the fixed position of the haptic at this point, we moved the haptic on one side outward by 1, 3, and 5 mm, respectively, while taking care not to shift the haptic position from here. iOCT and CALLISTO photography were used for the analysis of IOL tilt and decentration at each time-point, respectively (Fig. 2D).

Each of the above measurements were performed five times using the IOL (NX-70 + 20.0D), and the values were used in the analysis.

### The method of IOL tilt and decentration evaluation

For clinical evaluation, IOL images in vertical and horizontal directions were taken by using iOCT (RESCAN 700; Carl Zeiss Meditec) at the end of the surgery and swept-source anterior segment optical coherence tomography (AS-OCT) (SS-1000 CASIA; Tomey Corporation, Nagoya, Japan) during the follow-up period. For experimental evaluation, IOL images in vertical and horizontal directions were taken by using iOCT (RESCAN 700).

**Figure 3:**
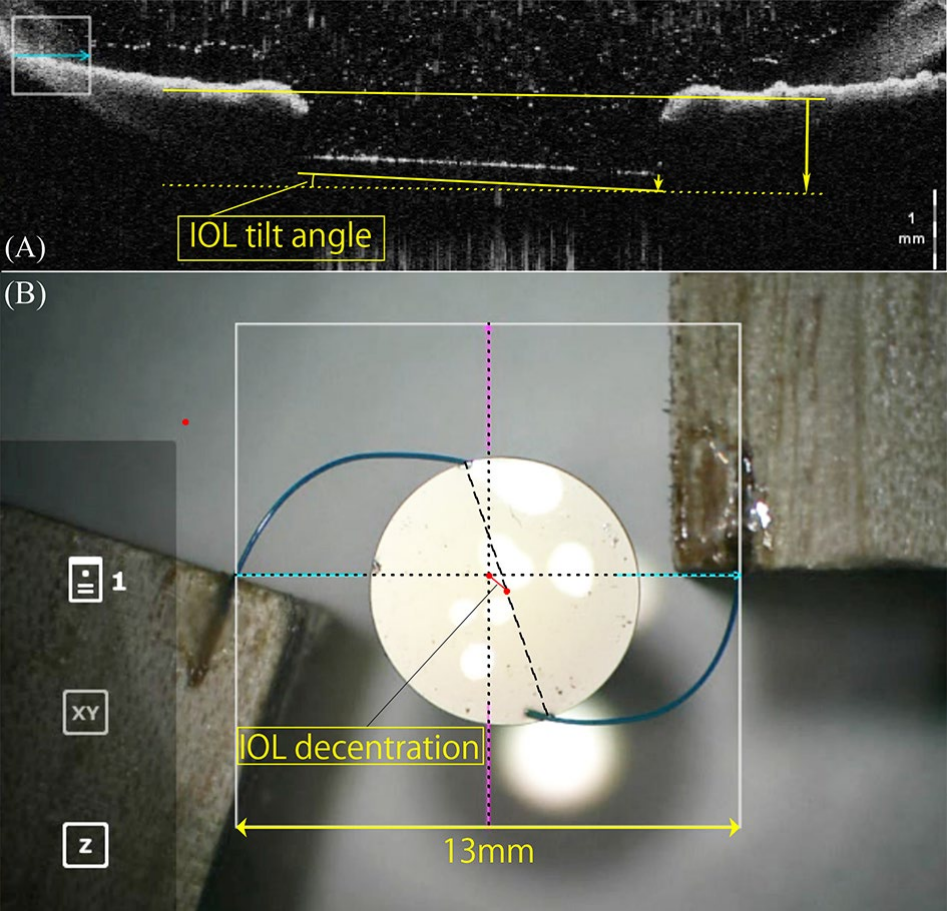
The OCT images that we used to calculate the IOL tilt in clinical evaluation (**A**). We took the reference line to be the line joining the straight line though the iris stroma on both sides. The angle between the reference line and the IOL anterior surface line was defined as the IOL tilt angle. The RESCAN700 images that we used to calculate the IOL decentration in experimental evaluation (**B**). The midpoints of the lines (top, bottom, left, and right) were connected using the line tool (dotted lines), and the intersection of the two midlines was set as the center of the frame. Then, the glued parts of the IOL haptics were connected with a straight line(dashed line) , and the midpoint of the line were regarded as the center of the IOL. The length of the measured line segment, or decentration distance (mm), was calculated from the ratio of the length of the line segment to the length of the frame line (pt), which corresponds to 13 mm. IOL = intraocular lens; OCT = optical coherence tomography.

For the evaluation of IOL tilt, image software (Adobe Photoshop®: Adobe, California, USA) was used. IOL images were imported in jpeg format, and the images were enlarged with the magnification tool (”Fit on screen”). Then, the reference line was taken. For clinical evaluation, the line was drawn to be the line joining the straight line though the iris stroma on both sides by using the “line tool”, and a similar line was drawn along the anterior surface of the IOL using the same tool. The angle between reference line and the IOL anterior surface line was defined as the IOL tilt angle(Fig. 3A) . In the experimental evaluation, the horizontal direction of the screen was assumed to be the reference line, and the inclination of the IOL anterior surface line was measured as the IOL tilt angle. The mean of the IOL tilt angle in the horizontal and vertical directions was defined as the mean IOL tilt angle, which was used in the analysis.

For the measurement of IOL decentration in experimental evaluation, the image software (Adobe Photoshop®: Adobe, California, USA) was used. IOL images were imported in jpeg format and enlarged with the magnification tool (”Fit on screen”). The midpoints of the lines (top, bottom, left, and right) were connected using the line tool, and the intersection of the two midlines was set as the center of the frame. Then, the glued parts of the IOL haptics were connected with a straight line, and the midpoint of the line were regarded as the center of the IOL. The length of the measured line segment, or decentration distance (mm), was calculated from the ratio of the length of the line segment to the length of the frame line (pt), which corresponds to 13 mm. The IOL decentration distance (mm) was calculated from the decentration distance under the basic conditions and each condition(Fig. 3B) .

### Statistical methods

For all variables, we reported the mean value and the standard deviation (SD). BCVA was converted to logarithmic minimum angle of resolution (log MAR) for statistical analysis. Friedman’s test and Wilcoxon t-test with Bonferroni correction for post hoc test were performed to assess changes in BCVA, IOP, CECD, and IOL tilt and decentration. The Mann–Whitney U-test and Kruskal–Wallis test for continuous variables were used to compare the parameters between groups. Statistical analyses were performed using SPSS software (version 24.0; IBM Corporation, Armonk, NY, USA). Statistical significance was considered at *p *< 0.05.

## Results

### Clinical evaluation

Table 1 summarizes the perioperative demographic data of the patients. Overall, 30 men and 11 women were included in this study. The mean age was 70.0 ± 13.8 years. All patients were followed-up for 3 months after surgery. Mean preoperative BCVA (logMAR), BCVA at 1 month after surgery, and BCVA at 3 months after surgery were 0.25 ± 0.45, 0.13 ± 0.32, and 0.13 ± 0.33, respectively (*p *< 0.01). Mean preoperative IOP (mmHg), IOP at 1 month after surgery, and IOP at 3 months after surgery were 15.0 ± 5.2, 14.1 ± 5.2, and 14.1 ± 3.8, respectively (*p *= 0.11). Mean preoperative CECD (cells/mm^2^), CECD at 1 month after surgery, and CECD at 3 months after surgery were 2102.1 ± 638.3, 1807.1 ± 700.9, and 1746.7 ± 736.5, respectively (*p *= 0.11). Mean IOL tilt angle (°) at the end of surgery and 1 month and 3 months after surgery were 1.81 ± 1.15, 2.11 ± 1.32, and 2.10 ± 1.66, respectively (*p *= 0.46) (Table 2). The mean surgically induced astigmatism 3 months after surgery was 0.93 ± 0.79 diopters, whereas the mean refractive error from the predicted value 3 months after surgery was − 0.48 ± 0.75 diopter. No apparent intra- or postoperative complications occurred during the follow-up period.

**Table 1:** Perioperative demographic data.

	Before surgery	1 month	3 months	*p-*value
BCVA (logMAR)	0.25 ± 0.45	0.13 ± 0.32	0.13 ± 0.33	< 0.01
IOP (mmHg)	15.0 ± 5.2	14.1 ± 5.2	14.1 ± 3.8	0.11
CECD (cells/mm^2^)	2102.1 ± 638.3	1807.1 ± 700.9	1746.7 ± 736.5	0.11

*BCVA* Best-corrected visual acuity, *logMAR* Logarithm of the minimum angle of resolution.*CECD* Corneal endothelial cell density.

**Table 2:** IOL tilt angle.

	The end of surgery	1 month	3 months	*p-*value
Mean IOL tilt (°)	1.81 ± 1.15	2.11 ± 1.32	2.10 ± 1.66	0.46
Horizontal IOL tilt (°)	1.67 ± 1.32	2.06 ± 1.77	1.99 ± 2.09	0.05
Vertical IOL tilt (°)	1.96 ± 1.68	2.16 ± 1.94	2.20 ± 2.31	0.85

*IOL* Intraoculear lens.

### Experimental evaluation

**Figure 4:**
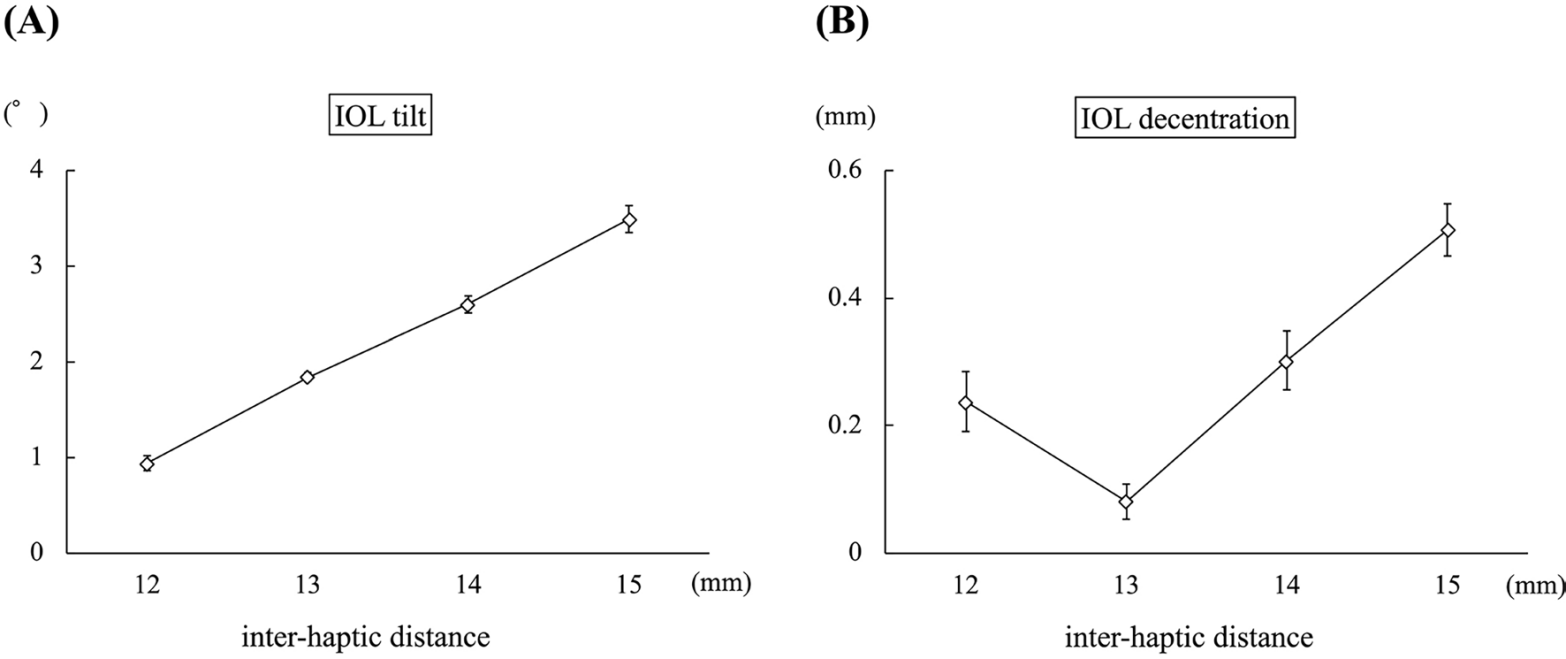
The graph shows the mean IOL tilt (**A**) and the IOL decentration (**B**) when the inter-haptic distance was 12 mm, 13 mm, 14 mm, and 15 mm.* IOL* Lntraocular lens.

**Figure 5:**
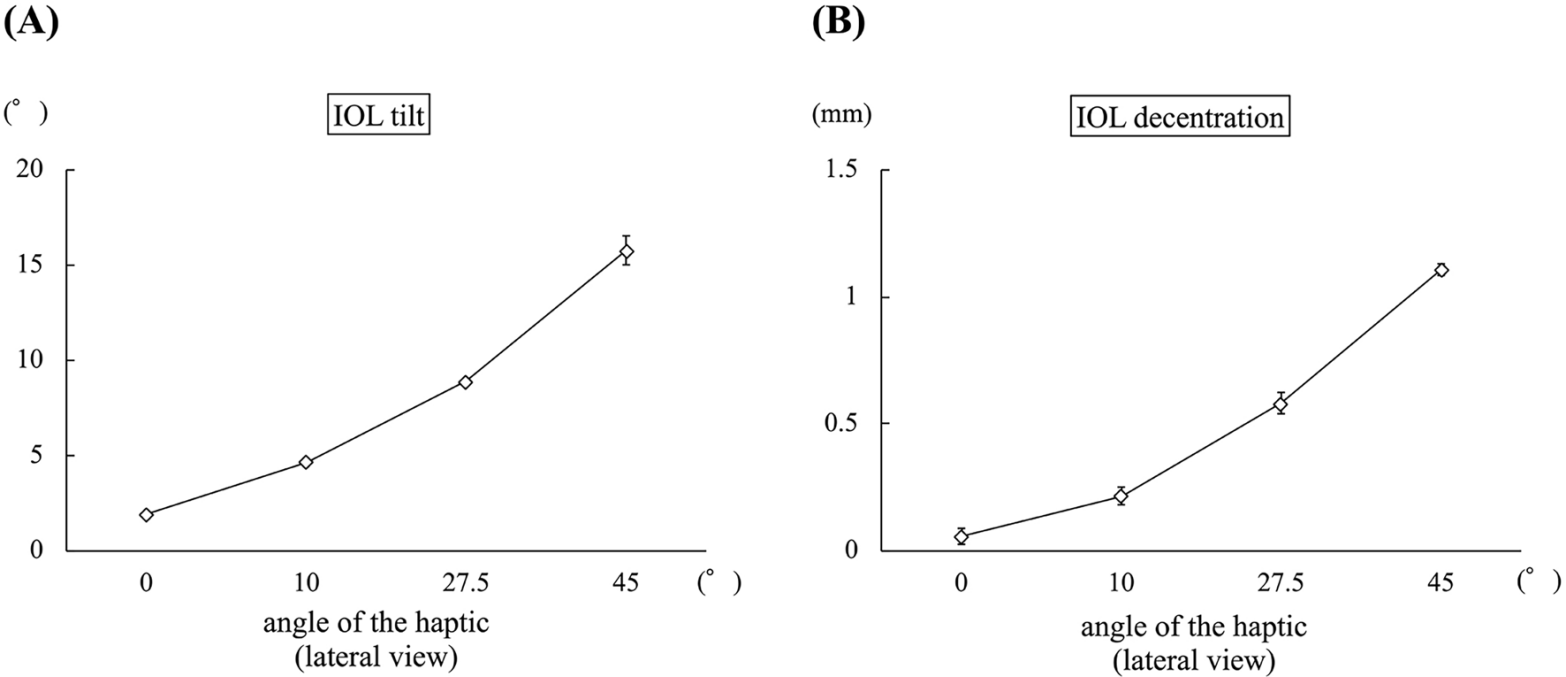
The graph shows the mean IOL tilt (**A**) and the IOL decentration (**B**) when the angle of the haptic was 0°, 10°, 27.5°, and 45° with observation from horizontal direction.* IOL* Lntraocular lens.

**Figure 6:**
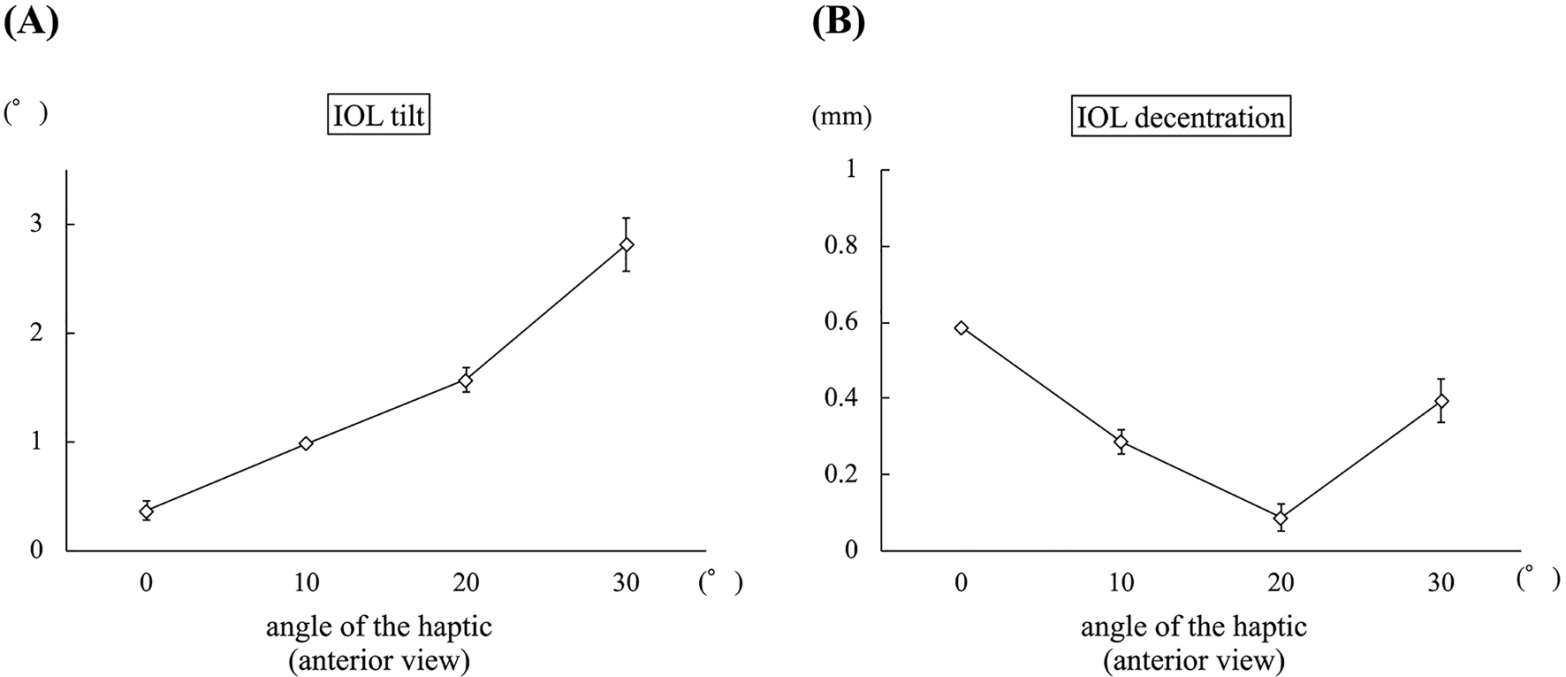
The graph shows the mean IOL tilt (**A**) and the IOL decentration (**B**) when the angle of the haptic was 0°, 10°, 20°, and 30° with observation from above. * IOL* Lntraocular lens.

**Figure 7:**
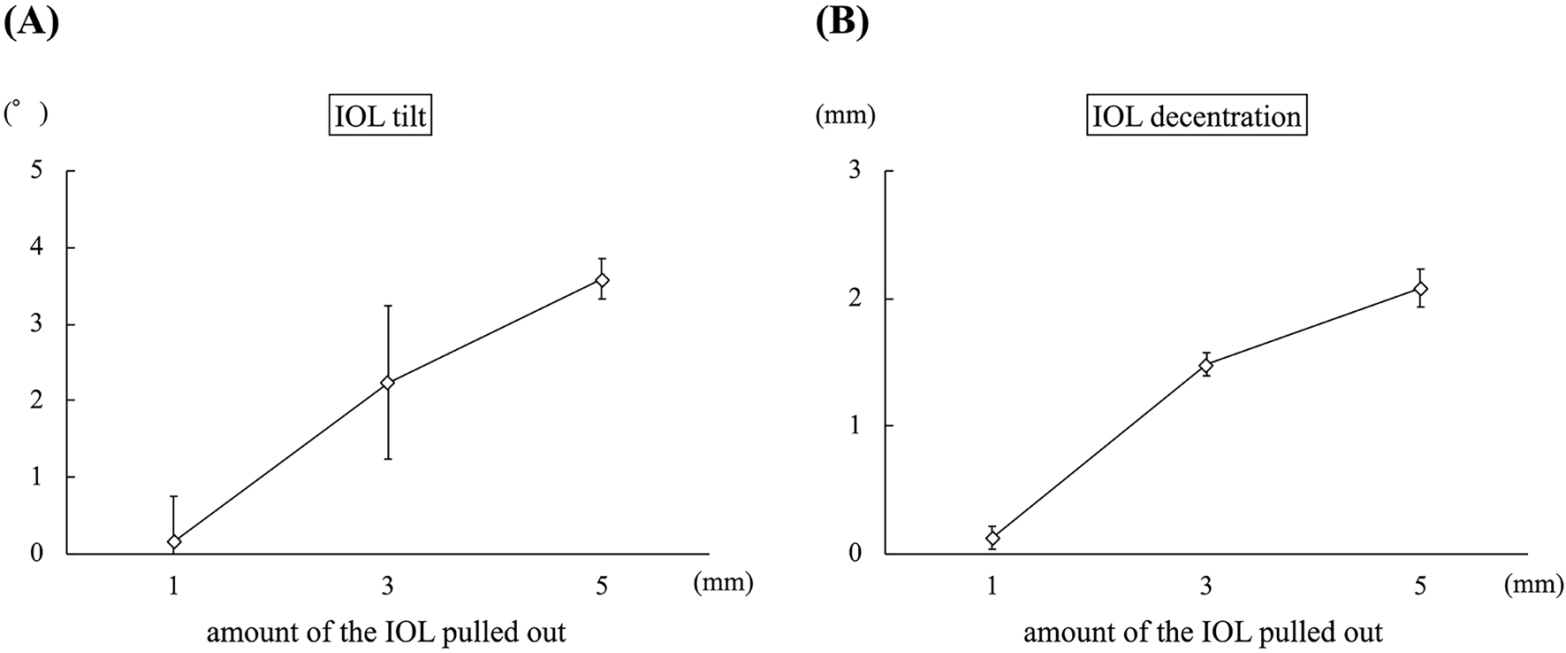
The graph shows the mean IOL tilt (**A**) and the IOL decentration (**B**) when the amount of IOL pulled out was 1 mm, 3 mm, and 5 mm. * IOL* Lntraocular lens.

In experiment 1, the mean IOL tilt angle (°) was 0.94 ± 0.09, 1.85 ± 0.05, 2.60 ± 0.10, and 3.49 ± 0.16 when the inter-haptic distance was 12 mm, 13 mm, 14 mm, and 15 mm, respectively (*p *< 0.01) (Fig. 4A). The IOL decentration (mm) was 0.24 ± 0.05, 0.08 ± 0.03, 0.30 ± 0.05, and 0.51 ± 0.05 when the inter-haptic distance was 12 mm, 13 mm, 14 mm, and 15 mm, respectively (*p *< 0.01) (Fig. 4B). In experiment 2, the mean IOL tilt angle (°) was 1.94 ± 0.09, 4.67 ± 0.11, 8.90 ± 0.11, and 15.78 ± 0.85 when the angle of the haptic was 0°, 10°, 27.5°, and 45°, respectively (*p *< 0.01) (Fig. 5A). The IOL decentration (mm) was 0.06 ± 0.03, 0.22 ± 0.04, 0.58 ± 0.05, and 1.11 ± 0.03 when the angle of the haptic was 0°, 10°, 27.5°, and 45°, respectively (*p *< 0.01) (Fig. 5B). In experiment 3, the mean IOL tilt angle (°) was 0.37 ± 0.10, 0.99 ± 0.02, 1.57 ± 0.13, and 2.81 ± 0.27 when the angle of the haptic was 0°, 10°, 20°, and 30°, respectively (*p *< 0.01) (Fig. 6A). The IOL decentration (mm) was 0.59 ± 0.01, 0.29 ± 0.04, 0.09 ± 0.04, and 0.40 ± 0.06 when the angle of the haptic was 0°, 10°, 20°, and 30°, respectively (*p *< 0.01) (Fig. 6B). In experiment 4, the mean IOL tilt angle (°) was 0.16 ± 0.59, 2.24 ± 1.00, and 3.59 ± 0.26 when the amount of the IOL pulled out was 1 mm, 3 mm, 5 mm, respectively (*p *< 0.01) (Fig. 7A). The IOL decentration (mm) was 0.13 ± 0.09, 1.49 ± 0.10, and 2.08 ± 0.16 when the amount of the IOL pulled out was 1 mm, 3 mm, and 5 mm, respectively (*p *< 0.01) (Fig. 7B).

## Discussion

For clinical evaluation, we performed a follow-up study on the real-time, intraoperative adjustment technique for IOL tilt by using iOCT during the Yamane intrascleral IOL fixation, which was first reported by Fukumoto^15^. In Fukumoto’s study, the IOL tilt was 4.58° ± 1.86° at 1 month after surgery, which was similar to the results of the present study (2.11° ± 1.32° at 1 month after surgery and 2.10° ± 1.66° at 3 months after surgery). In contrast, Yamane, et al. reported that the postoperative IOL tilt was 3.4° ± 2.5° at 36 months after surgery without the usage of iOCT in their first report about flanged intrascleral IOL fixation^16^. In several studies without the use of iOCT, the postoperative IOL tilts were 8.80° ± 3.86°, 3.52° ± 3.00°, and 4.32° ± 3.12° at the end of the follow-up period^17–19^. These results show that the postoperative mean IOL tilt was not different between the technique with/without correcting IOL tilt in real time by means of the iOCT. However, the standard deviation of the IOL tilt seems to be much smaller than that in previous reports without iOCT. In addition, in the case of successful intracapsular IOL implantation, the postoperative IOL tilt has been reported to be 5.21° ± 1.47°^17^. This result shows that both the postoperative IOL tilt and its standard deviation were comparable between the present study and usual intracapsular IOL fixation. Additionally, Kimura reported that even in normal eyes, the crystalline lens exhibited an average tilt of 5.25° under mydriatic conditions, while the normally inserted IOL also had an average tilt of 4.65° under the same conditions^20^. These results align with our own findings. These collective results suggest that real-time IOL tilt correction using iOCT has the potential to further reduce IOL tilt in all subjects, thus preventing significant unintended tilt. As a result, this approach offers the possibility of achieving IOL tilt levels similar to those seen in a physiological crystalline lens, even in the absence of a normal crystalline lens capsule.

In this study, the postoperative refractive difference from the predicted value was also investigated, and it was found to be − 0.48 ± 0.75 diopters 3 months after the surgery. On the other hand, the postoperative refractive difference in the three previous reports with flange method were − 0.21 ± 0.99^16^, 0.86 ± 6.59^17^, and 0.03 ± 0.93^18^ diopters, and the results of our study tend to have higher postoperative refractive difference. In these three previous reports, a fixed value of 2 mm was used for the distance of the scleral insertion site of the 30-gauge needle from the limbus. As the distance from the limbus was constant, it was probable that postoperative refractive difference from the predicted value would not readily occur if there was no IOL tilt. In our study, the scleral insertion site of the 30-gauge needle was determined using a Yamane double-needle guide^13^. As the needle insertion angle is constant, and inconsistency in the distance from the limbus does not readily develop, this offers the advantage of suppressing the postoperative IOL tilt. On the other hand, depending upon the ocular axial length and the corneal diameter, the distance of the insertion site from the limbus is not constant because the size of stabilizer is constant. It is possible that this inconstancy affected the final induction of postoperative refraction difference. These findings suggest that, for insertion of a 30-gauge needle, in order to suppress postoperative IOL tilt and refractive difference, it may be important to insert the needle at a constant angle and set constant values for the distance of insertion from the limbus.

Clinical experience and the above results of clinical evaluation support the hypothesis that insertion angle and position are important to minimize postoperative IOL tilt, but which of the various angular and positional factors are particularly important to prevent postoperative IOL tilt and decentration are still unknown. To clarify these clinical questions, an experimental evaluation of the factors affecting IOL tilt and decentration after intrascleral IOL fixation using the flange method was also performed using iOCT system similar to that detailed above.

We found that all four of the parameters were linked with IOL tilt and decentration, and in particular, deviation in the insertion angle of the 30-gauge needle in the vertical direction had a major influence. This finding is in good agreement with our clinical experience and is considered to constitute useful data not only for physicians who are learning how to perform intrascleral IOL fixation, but also for those who already have considerable experience. Of the four parameters, three parameters other than amount of haptics pulled-out were difficult to correct after insertion of a 30-gauge needle, suggesting the importance of correct insertion during surgery. However, as the surgeon determines the insertion angle while observing the eyeball from the front, that is, from the corneal side during surgery, it is particularly difficult to perform surgery with an accurately defined insertion angle as viewed laterally. Thus, especially when the surgery is performed by inexperienced surgeons, stabilizers should be used, and other measures including iOCT should be taken to ensure that the insertion angle is constant.

The ability to adjust the amount of haptic pulled-out after insertion is one of the key techniques to minimize IOL tilt as clinically possible^11^. However, our results suggest that the IOL tilt that can be adjusted by the amount of haptic pulled-out has limits and is not very effective. Additionally, the uneven pulled-out amount has been found to increase the amount of IOL decentration. Furthermore, the extension of the IOL for surgical intrascleral fixation can alter the higher-order aberrations of IOL^21^. This indicates that it is important to set the angle and position correctly during insertion without resorting to this procedure.

The limitations of this study are as follows. First, the clinical evaluation has a small sample size and is a retrospective study. Additionally, a control group without the use of iOCT was not established. It is necessary to increase the number of cases and conduct a prospective study in the future. Furthermore, the experimental evaluation is based on model results and may not directly correlate with actual surgical outcomes. Therefore, it is important to understand that the results of the experimental evaluation in this study may not directly match clinical experiences. However, this study provides a detailed analysis of one of the issues associated with the rapidly spreading Yamane intrascleral IOL fixation technique, such as postoperative unexpected large IOL tilt, and holds significant value.

In conclusion, we reported the utility of iOCT for clinically minimizing postoperative IOL tilt and, as an experimental evaluation, assessed factors that contribute to IOL tilt. By making full use of the latest intraoperative examination equipment such as iOCT, it is possible to deepen our understanding of surgical techniques and improve surgical results.

## Supplementary Information

Supplementary Legends.

Supplementary Video 1.

Supplementary Video 2.

## Data Availability

The datasets generated during and/or analyzed during the current study are available from H. Imai, the corresponding author, on reasonable request.
