# Supplementary material for: Usefulness of intraoperative optical coherence tomography to minimize the intraocular lens tilt during the intrascleral fixation: a clinical and experimental evaluation
Source: Sci Rep. 2023 Jul 26;13:12065. doi: 10.1038/s41598-023-39294-0 (PMC10372065; doi:10.1038/s41598-023-39294-0)
Supplement: Supplementary file 1 — Supplementary Legends. [file 41598_2023_39294_MOESM1_ESM.docx]

**List of Supplemental Digital Content**

Supplemental Digital Content 1. Video demonstrating surgical procedure and the objective observation of the positional relationship between the IOL and iris.

Supplemental Digital Content 2. Video demonstrating the objective observation of the IOL tilt and decentration, and the method of moving the IOL haptics.
